# Regional Amyloid Deposition in Amnestic Mild Cognitive Impairment and Alzheimer's Disease Evaluated by [^18^F]AV-45 Positron Emission Tomography in Chinese Population

**DOI:** 10.1371/journal.pone.0058974

**Published:** 2013-03-14

**Authors:** Kuo-Lun Huang, Kun-Ju Lin, Ing-Tsung Hsiao, Hung-Chou Kuo, Wen-Chuin Hsu, Wen-Li Chuang, Mei-Ping Kung, Shiaw-Pyng Wey, Chia-Ju Hsieh, Yau-Yau Wai, Tzu-Chen Yen, Chin-Chang Huang

**Affiliations:** 1 Department of Neurology and Dementia Center, Chang Gung Memorial Hospital and Chang Gung University, Taoyuan, Taiwan; 2 Molecular Imaging Center and Department of Nuclear Medicine, Chang Gung University and Chang Gung Memorial Hospital, Taoyuan, Taiwan; 3 Healthy Aging Research Center and Department of Medical Imaging and Radiological Sciences, College of Medicine, Chang Gung University, Taoyuan, Taiwan; 4 Department of Radiology, University of Pennsylvania, Pennsylvania, United States of America; 5 Department of Radiology, Chang Gung Memorial Hospital, Taoyuan, Taiwan; University of Manchester, United Kingdom

## Abstract

**Background:**

To compare the neocortical amyloid loads among cognitively normal (CN), amnestic mild cognitive impairment (aMCI), and Alzheimer's disease (AD) subjects with [^18^F]AV-45 positron emission tomography (PET).

**Materials and Methods:**

[^18^F]AV-45 PET was performed in 11 CN, 13 aMCI, and 12 AD subjects to compare the cerebral cortex-to-whole cerebellum standard uptake value ratios (SUVRs) of global and individual volumes of interest (VOIs) cerebral cortex. The correlation between global cortical [^18^F]AV-45 SUVRs and Mini-Mental State Examination (MMSE) scores was analyzed.

**Results:**

The global cortical [^18^F]AV-45 SUVRs were significantly different among the CN (1.08±0.08), aMCI (1.27±0.06), and AD groups (1.34±0.13) (p = 0.0003) with amyloidosis positivity rates of 9%, 62%, and 92% in the three groups respectively. Compared to CN subjects, AD subjects had higher SUVRs in the global cortical, precuneus, frontal, parietal, occipital, temporal, and posterior cingulate areas; while aMCI subjects had higher values in the global cortical, precuneus, frontal, occipital and posterior cingulate areas. There were negative correlations of MMSE scores with SUVRs in the global cortical, precuneus, frontal, parietal, occipital, temporal, posterior cingulate and anterior cingulate areas on a combined subject pool of the three groups after age and education attainment adjustment.

**Conclusions:**

Amyloid deposition occurs relatively early in precuneus, frontal and posterior cingulate in aMCI subjects. Higher [^18^F]AV-45 accumulation is present in parietal, occipital and temporal gyri in AD subjects compared to the aMCI group. Significant correlation between MMSE scores and [^18^F]AV-45 SUVRs can be observed among CN, aMCI and AD subjects.

## Introduction

Alzheimer's disease (AD) is the most common cause of dementia and leads to progressive dysfunctions in memory and other cognitive domains. The diagnosis of AD is hampered by a lack of noninvasive biomarkers. Moreover, 67% of dementia patients with mild clinical symptoms may not be diagnosed by physicians [1]. The most widely accepted pathogeneses of AD include increased production or impaired clearance of amyloid-beta oligomers, resulting in excessive fibrillary amyloid plaque accumulation in the brain. Furthermore, the accumulation of amyloid plaques may precede the diagnosis of AD by 10 years [2]. Therefore, a reliable biomarker for determination of amyloid plaque aggregates could facilitate an early diagnosis and treatment of AD.

Molecular neuroimaging modalities such as positron emission tomography (PET) have a potential to provide functional information on the pathological process of AD. Several PET ligands have been investigated to determine their ability for detecting amyloid plaques, including [^11^C]PIB [3], [^11^C]SBI-13 [4], [^11^C]BF-227 [5], [^11^C]MeS-IMPY [6], [^18^F]FDDNP [7], [^18^F]AV-45 (florbetapir) [8], [^18^F]AV-1 (florbetaben, BAY94-9172) [9], and [^18^F]GE067 (flutemetamol) [10]. At present, the most widely researched imaging technique utilizes [^11^C]PIB to detect amyloid plaques, but the short half-life of the carbon-11 (approximately 20.4 min) of [^11^C]PIB limits its clinical application. On the other hand, PET tracers utilizing fluorine-18 have been designed to overcome this limitation since this isotope has a longer half-life of about 109.4 min, which makes off-site preparation and regional distribution easier. Among these tracers, [^18^F]AV-45 has gained great attention as a PET imaging agent targeting amyloid plaques and been selected as the standard plaque imaging agent for the Alzheimer's Disease Neuroimaging Initiative (ADNI) [11]. [^18^F]AV-45 shows good amyloid-binding affinity with a desirable pharmacokinetics. It remains at steady levels in the brain 30–90 min after injection, and a short imaging time (5–10 min) is sufficient [8]. A recent phase III study with 35 terminally ill participants was reported to compare [^18^F]AV-45 imaging and postmortem amyloid immunohistochemistry. The results showed that [^18^F]AV-45 imaging is well correlated with postmortem amyloid immunohistochemistry [12].

In this preliminary study, we aimed to compare the amyloid plaque distribution on [^18^F]AV-45 PET scan in cognitively normal (CN), amnestic mild cognitive impairment (aMCI) and AD subjects, and correlate the cerebral cortical amyloid load with cognitive functioning among CN, aMCI and AD subjects. In addition, we aimed to determine a cutoff value to differentiate CN subjects from AD subjects in a Chinese population.

## Methods

### Participants

Thirty-six subjects were studied at the Center for Dementia of the Linkou Chang Gung Memorial Hospital (CGMH), Taiwan, including 11 CN, 13 aMCI, and 12 AD subjects. All subjects underwent a neurological examination, neurocognitive evaluation, and routine blood analysis. Study subjects were categorized on the basis of the consensus of panels composed of neurologists, neuropsychologists, neuroradiologists and experts in nuclear medicine. The CN subjects were volunteers with normal cognitive performance; the methods for CN subject recruitment have been described previously [8]. The diagnostic criteria for aMCI were based on those proposed by Petersen *et al* [13]: (1) subjective memory complaints by the patient or an informant, (2) relatively normal performance in other cognitive domains and (3) normal activities of daily living, (4) objective memory impairment on at least one neurocognitive test of memory performance, and (5) no dementia according to DSM-IV criteria [14]. No rigid cutoff score was applied to determine objective memory impairment, but aMCI was generally determined when memory measures fell 1.0–1.5 standard deviations below the means for age-matched norms in Taiwan. The diagnosis of AD was made when subjects fulfilled the National Institute of Neurological and Communicative Disorders and Stroke–Alzheimer's Disease and Related Disorders Association (NINCDS-ADRDA) criteria for probable AD [15]. A comprehensive battery of neurocognitive tests was administered to obtain objective evidence of cognitive impairment, including the Mini-Mental State Examination (MMSE) [16], Clinical Dementia Rating (CDR), logical memory in the Wechsler memory scale-revised (LM), visual-association memory test (VAMT), category verbal fluency test (CVFT), trail-making A test (TMAT), and clock-drawing test (CDT) [17]. Prior to enrollment, the study objectives and protocol were well informed to all subjects and also their next of kin, caregivers or guardians if subjects were clinically suspected of AD. The written informed consent was obtained from all subjects before their participation in the study. In addition, the next of kin or guardians of 6 AD patients also gave their written informed consents since these patients' capacity to comprehend the study protocol was compromised or they could not sign their names clearly. The study protocol and the procedure of obtaining informed consents were approved by the institutional review board of Chang Gung Memorial Hospital and the Governmental Department of Health.

### Magnetic Resonance Imaging (MRI) Acquisition

The MRI images were acquired on a 3T MR scanner (Magnetom Trio, a TIM system; Siemens, Erlangen, Germany). The scanning protocol included an axial fluid attenuation inversion recovery (FLAIR) sequence (TR = 9000 ms, TE = 87 ms, TI = 2500 ms, voxel size = 0.9×0.7×4 mm^3^) and a whole brain axial three-dimensional T1-weighted magnetization prepared rapid acquisition gradient echo (MP-RAGE) sequence (TR = 2000 ms, TE = 2.63 ms, TI = 900 ms, flip angle = 9°, voxel size = 1×1×1 mm^3^), which was subsequently reformatted as planes perpendicular to the long axis of the hippocampus in 2 mm slice thickness. An additional coronal T2-weighted turbo spin echo sequence (TR = 7400 ms, TE = 95 ms, voxel size = 0.4×0.4×2 mm^3^) was acquired with the identical geometric orientation with the reformatted coronal T1-weighted images [8].

### [^18^F]AV-45 PET Imaging

[^18^F]AV-45 was synthesized at the cyclotron facility of Chang Gung Memorial Hospital following the method described previously [18]. In brief, [^18^F]fluoride was obtained by bombardment of ^18^O-enriched water with 12-MeV protons accelerated in a SHI-12S cyclotron (Sumitomo Heavy Industries, Tokyo, Japan). The [^18^F]fluoride was then trapped on an Accel Plus QMA cartridge (Waters (Milford, MA) and eluted to a reaction vessel using a mixture of K_2_CO_3_, Kryptofix2.2.2 and acetonitrile. Following azeotropic distillation, the dehydrated [^18^F]fluoride was reacted with AV-105, the precursor for synthesis of [^18^F]AV-45 provided by Avid Radiopharmaceiticals (Philadelphia, PA), to form a fluorinated intermediate, N-Boc-[^18^F]AV- 45, which was in turn to be hydrolyzed using HCl to remove the protection group. Product [^18^F]AV- 45 was obtained after purification with semi-preparative HPLC. The radiochemical purity of [^18^F]AV-45 was greater than 95%, and the specific activity was greater than 4000 TBq/mmol at the end of synthesis (EOS). The mean administered activity was 365 MBq (range, 325–394 MBq). The PET acquisition protocol and optimal scanning time were adapted from previous reports [8], [19]. In brief, helical computed tomography images were obtained for attenuation correction at 40 min. Each PET acquisition consisted of two 5-min dynamic frames obtained 50–60 min post-injection in 3D mode [19] from the Biograph mCT PET/CT System (Siemens Medical Solutions, Malvern, PA, USA). PET projection data were iteratively reconstructed using 3-D OSEM algorithm of 4 iterations, 24 subsets, postsmoothed by a Gaussian filter of 2 mm FWHM and zoom = 3, and with CT-based attenuation correction. Scatter and random correction were also performed using the correction methods provided by the manufacture. The reconstructed images were with pixel size of 0.679 mm, and slice thickness of 2.027 mm. Individual frames of the [^18^F]AV-45 PET dynamic series were realigned if motion was detected. Summed images were subsequently created for analysis [20].

### [^18^F]AV-45 Imaging Processing and Atlas-based Quantitative Volume of Interest (VOI) Analysis

Both PET and MRI images from each subject were co-registered first, and then MRI images were spatially normalized into the MNI T1 template as in Statistical Parametric Mapping 5 (SPM5) (Wellcome Department of Cognitive Neurology, University College London, London, UK). The transformation parameters of MR normalization were then applied to corresponding PET images. All the image data were processed in the PMOD software (version 3.2; PMOD Technologies Ltd., Zurich, Switzerland). For all subjects, a binary gray matter mask was first created by taking the non-zero pixels from an averaged gray matter probability map generated from the segmentation of the spatially normalized MR images from normal subjects. The final VOI template for VOI analysis was then obtained by taking the intersection of both the binary gray matter mask and the automated anatomic labeling (AAL) atlas (VOI template) for all subjects [21], [22]; this step was performed to minimize the inclusion of both cerebrospinal fluid and white matter (and thus non-specific white matter [^18^F]AV-45 retention) in the statistical measures of all VOIs [23]. The original AAL atlas which contains 116 regions was combined into the following 8 labeled VOIs for further analysis: the precuneus, frontal, parietal, occipital, temporal, posterior cingulate and anterior cingulate areas, and the whole cerebellum. The standard uptake value ratio (SUVR) was calculated by determining the ratios of the integrated activities between the target VOI (cortical regions) and whole cerebellum reference VOI.

Voxel-based statistical analysis was conducted with SPM5 [24]. The spatial normalization of individual [^18^F]AV-45 images to MNI MRI T1-template space was performed in the same way as that for the VOI analysis described above. All voxels in the normalized [^18^F]AV-45 images were divided by the mean [^18^F]AV-45 uptake of the whole cerebellum VOI in each subject to form SUVR images. Moreover, all PET images in SPM analysis were smoothed by 8 mm FWHM Gaussian filtering. Voxel-wise [^18^F]AV-45 uptake differences among the CN, aMCI, and AD subjects were assessed in SPM5. Statistical maps displaying group differences were shown at a significance value of p<0.01 and extent voxels of 100. Due to a relatively small sample size of our study, and to increase the sensitivity in the SPM analysis, the lower threshold of two-tailed p<0.01 under uncorrected statistics was applied here [25].

### Determination of the Threshold for Cerebral Cortical Amyloidosis in [^18^F]AV-45 Images

To quantitatively measure the threshold for global cerebral cortical amyloid burden, the [^18^F]AV-45 images of the AD and CN groups were compared to derive the cutoff point with receiver operating characteristic (ROC) analysis. Subjects with a cortical SUVR level exceeding the threshold were categorized as positive for cerebral amyloidosis. In our cases, the threshold derived from the AD and CN groups was 1.178 with the area under the curve of 0.96 (p<0.001); the sensitivity and specificity were 92% and 91%, respectively.

### Statistical Analyses

Data were expressed as means±SD or absolute numbers with proportions for descriptive statistics. Continuous variables were analyzed by non-parametric statistics with the Mann–Whitney *U*-test. Categorical data were analyzed using Fisher's exact test. Differences of [^18^F]AV-45 cortical SUVRs between groups were assessed by non-parametric one-way analysis of variance (ANOVA) using the Kruskal–Wallis test followed by Dunn's post hoc multiple comparison test. Correlations between cognitive test scores and cortical SUVRs were analyzed with both Pearson correlations and partial regression with adjustment for age and years of education. Statistical analyses were performed with SAS version 9.2 (SAS Institute Inc., New York, USA), and p<0.05 was considered significant.

## Results

The study recruited 11, 13, and 12 subjects in the CN, aMCI, and AD groups, respectively. The demographic data were shown in Table 1. None of the subjects had adverse events directly related to [^18^F]AV-45 administration.

**Table 1:** Demographic characteristics of the CN, aMCI, and AD groups.

	CN (n = 11)	aMCI (n = 13)	AD (n = 12)	p
Gender (M/F)	6/5	5/8	3/9	0.35
Age, years	69.3 (6.6)	70.0 (11.2)	75.8 (5.7)	0.15
CDR	0.0 (0.0)	0.5 (0.0)*	1.1 (0.3)*	<0.0001
MMSE	27.9 (1.6)	19.7 (2.4)*	12.9 (3.7)*	<0.0001
Memory deficit duration, years	-	3.0 (1.2)	5.9 (2.2)	<0.001
Education level, years	9.4 (4.1)	6.2 (4.1)	7.2 (4.3)	0.19

* p<0.05 compared to the CN group.

Values were mean (SD) or numbers of patients.

CN, cognitively normal; aMCI, amnestic mild cognitive impairment; AD, Alzheimer's disease; CDR, clinical dementia rating; MMSE, mini-mental state examination.

### VOI Analysis

**Figure 1:**
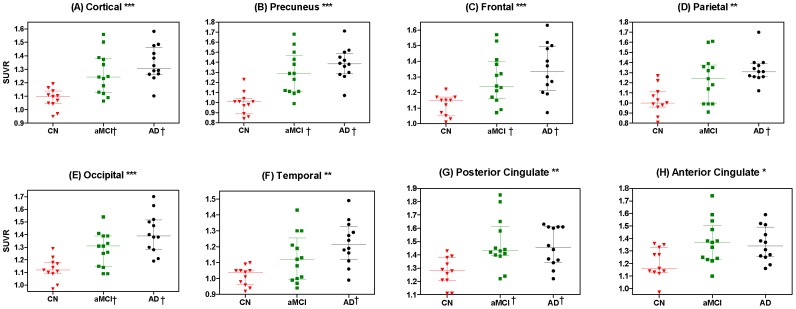
Scatter plots showing general and individual region-to-cerebellum ratios between different groups. Comparisons of [^18^F]AV-45 SUVR in the predefined VOIs of the global cortical (A), precuneus (B), frontal (C), parietal (D), occipital (E), temporal (F), posterior cingulate (G) and anterior cingulate (H) areas among the cognitively normal (CN), amnestic mild cognitive impairment (aMCI), and Alzheimer's disease (AD) subjects. (* *p*<0.05, ** *p*<0.005, *** *p*<0.0005, when comparing SUVR among CN, aMCI and AD subjects with the Kruskal–Wallis ANOVA test. † *p*<0.05 versus CN subjects with Dunn's post hoc analysis).

**Figure 2:**
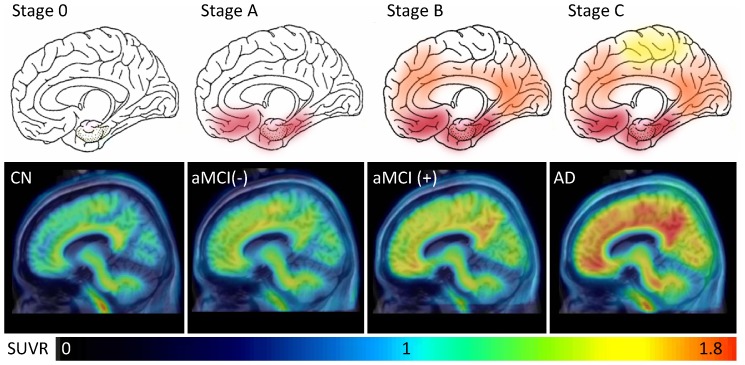
Representative sagittal [^18^F]AV-45 PET images. Varying degrees of [^18^F]AV-45 PET uptake in CN, aMCI negative for cerebral amyloidosis (aMCI (-)), aMCI positive for cerebral amyloidosis (aMCI (+)), and AD subjects (bottom row) were closely corresponding to the pathological amyloid deposition distribution adapted from Braak and Braak (top row) [26].

The VOI analysis of [^18^F]AV-45 images revealed significant differences in the global cortical SUVR among subjects with CN, aMCI and AD (Figure 1). Regarding to the differences in regional SUVRs, the precuneus, frontal, parietal, occipital, temporal, and posterior and anterior cingulate cortical SUVRs were also different among the three groups. In the post hoc analyses, all of these areas except the anterior cingulate had a higher SUVR of [^18^F]AV-45 in AD subjects than CN subjects. Furthermore, the SUVRs of the global cortical, precuneus, frontal, occipital and posterior cingulate areas were higher in aMCI subjects than CN subjects. There was no difference in [^18^F]AV-45 SUVRs of these predefined areas between aMCI and AD subjects. With the SUVR cutoff point of 1.178, the positive rate of cerebral cortical amyloidosis was 8%, 62%, and 92% for CN, aMCI and AD groups, respectively. Interestingly, there were varying degrees of [^18^F]AV-45 retention in CN, aMCI negative for cerebral amyloidosis (aMCI (−)), aMCI positive for cerebral amyloidosis (aMCI (+)), and AD subjects, whose patterns closely followed the amyloid deposition distribution observed in post mortem brain tissue (Figure 2) [26].

### SPM Analysis

**Figure 3:**
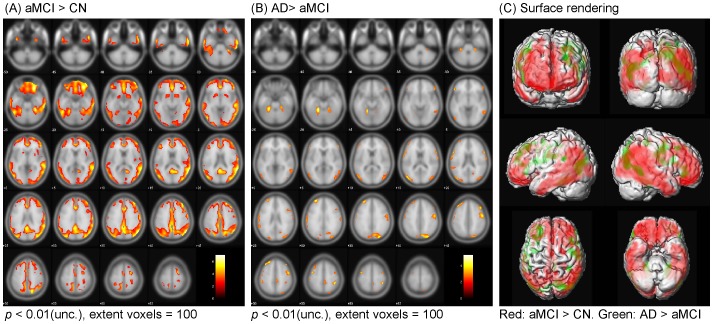
Statistical parametric mapping analysis: Localization of increased [^18^F]AV-45 retention between CN, aMCI and AD subjects. Comparisons of [^18^F]AV-45 SUVRs between cognitively normal (CN) and amnestic mild cognitively impairment (aMCI) subjects (A), and between aMCI and Alzheimer's disease (AD) subjects (B) (Uncorrected for multiple comparisons and the color bar values indicate the value of the T-statistic in each display). Surface rendering was used to illustrate the cortical areas where [^18^F]AV-45 SUVRs were increased in aMCI than CN subjects (red) and increased in AD than aMCI subjects (green) (C).

The SPM analysis showed that aMCI subjects had significantly higher [^18^F]AV-45 uptake than CN subjects in the frontal, temporal, parietal, occipital areas, as well as in the precuneus, which showed the most prominent differences (Figure 3A). These results were supported by the automated VOI analysis in which aMCI patients showed 15% increased [^18^F]AV-45 uptake in the frontal cortex (p<0.05) and 30% in the precuneus (p<0.005) than the CN group. Individually, in the precuneus, 8 of 13 aMCI subjects had [^18^F]AV-45 uptake values above 2 SD from the CN group mean. Comparing the SUVRs between AD and aMCI subjects, AD subjects had a slightly increased [^18^F]AV-45 uptake over the frontal, temporal and parietal regions (Figures 3B and 3C). Correspondingly, the VOI analysis showed significant increase of [^18^F]AV-45 in AD as compared to CN subjects in the additional brain regions including parietal (30% increase from the CN group mean, p<0.01), occipital (26% increase from the CN group mean, p<0.01), and temporal regions (20% increase from the CN group mean, p<0.01). Tables S1, S2 and S3 listed the peaks of the most significant increase in [^18^F]AV-45 uptake obtained in this analysis with Talairach and Tournoux coordinates, x, y, and z in millimeters of these peaks, as well as the corresponding z scores.

### Correlations between [^18^F]AV-45 SUVRs and Cognitive Function

**Figure 4:**
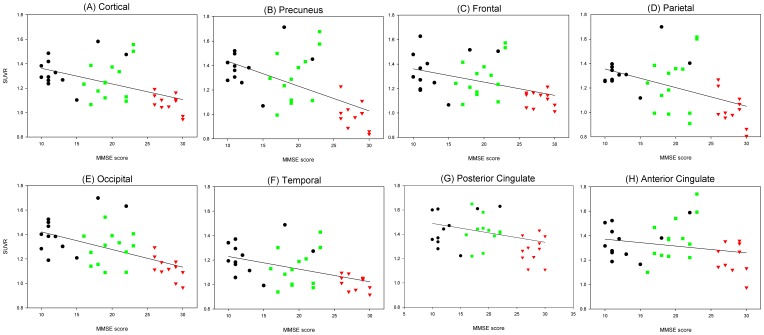
Relationship between MMSE scores and global and regional cortical [^18^F]AV-45 retention. Results of the negative regressions between MMSE scores and global and regional cortical [^18^F]AV-45 SUVRs among the cognitive normal, amnestic mild cognitive impairment, and Alzheimer's disease subjects with age and years of education as covariates.

There was no association of the global cortical [^18^F]AV-45 SUVR with age and education attainment. There was a negative correlation of the global cortical [^18^F]AV-45 SUVR with the scores of MMSE (adjusted R^2^ = 0.44), LM (adjusted R^2^ = 0.44), CVFT (adjusted R^2^ = 0.37), TMAT (adjusted R^2^ = 0.48), CDT (adjusted R^2^ = 0.56) and VAMT (adjusted R^2^ = 0.57) with all p values <0.001 after adjustment for age and education. Furthermore, there was a negative correlation of MMSE scores with all the predefined VOI areas after adjustment for age and education (Figure 4).

## Discussion

Although the cause of AD is still unknown, the presence of amyloid plaques plays a pivotal role in the pathogenesis of the disease. Studies show that the presence of amyloid plaques in patients with mild cognitive impairment may increase the potential of conversion to AD [27]. Therefore, the development of imaging techniques to detect amyloid plaques in vivo would be helpful for early diagnosis and monitoring disease progress [28]. Several fluorine-18-labeled radiotracers, including [^18^F]AV-45 (florbetapir), [^18^F]AV-1 (florbetaben) and [^18^F]GE067 (flutemetamol), have been extensively employed for amyloid plaque detection both in vivo and ex vivo [29]. Although [^11^C] PIB PET has been applied to compare the amyloid plaque distribution among CN, MCI and AD subjects in the Asian population [30], there are limited reports about fluorine-18-labeled amyloid PET studies in non-Caucasians [20], [31], [32]. In a recent report by Barthel *et al*, the Caucasian elderly normal subjects had higher SUVRs in different neocortical regions as compared to the Japanese elderly normal subjects on the florbetaben PET [31]. To our knowledge, this is the first [^18^F]AV-45 PET study to compare the amyloid loads among CN, aMCI, and AD subjects in vivo in an Eastern population. We found that the [^18^F]AV-45 SUVR was markedly increased in aMCI and AD subjects compared to CN subjects, regardless of whether it was measured with the VOI or SPM methods. In addition, we also applied the ROC method to determine the threshold for global cortical amyloid positivity, which was 1.178 with a sensitivity of 92% and a specificity of 91%, respectively. Our findings are concordant with those of Fleisher *et al* [33]. They applied the data from another post-mortem study to determine the [^18^F]AV-45 threshold for amyloid positivity, and the pathology-based threshold was 1.17, which is similar to our clinical diagnosis-based threshold of 1.178.

At present, the diagnosis of AD is mainly based on clinical manifestations, brain structure imaging, laboratory study, and neuropsychological assessment. Around 10–20% of clinically diagnosed AD patients do not have amyloid plaques on post-mortem pathological examination [34], [35]. In our study, the positivity of amyloidosis in the CN, aMCI, and AD groups was 9%, 62%, and 92%, respectively. The positivity for amyloidosis in AD patients (92%) is relatively high compared to the 80.9% observed by Fleisher *et al* [33]. One of the reasons is that our AD subjects had a relatively low cognitive performance and education attainment. Besides, the average disease duration was 5.9±2.2 years. Interestingly, 1 of the 12 AD patients (8%) had a relatively low global cortical SUVR, with a level similar to that of the CN subjects in our study, despite the fact that the patient exhibited a typical presentation of AD and fulfilled the most widely applied NINCDS-ADRDA criteria. However, the criteria may still allow a degree of uncertainty given the fact of the lack of pathological findings. On the other hand, PET radiotracers may fail to bind amyloid plaques because different fibrillary amyloid aggregate forms may have different affinities for amyloid radiotracers [36], [37].

The accumulation of amyloid plaques is a relentless process with a stereotypic progression pattern in the disease course of AD. In our study, the distribution of [^18^F]AV-45 uptake in the aMCI group was intermediate between that of the CN and AD groups. Comparing the regional [^18^F]AV-45 SUVRs between aMCI and CN subjects, a higher [^18^F]AV-45 SUVR was noted over the precuneus, frontal, occipital and posterior cingulate areas. These findings were similar with the report from Kempainen *et al* that aMCI patients had higher [^11^C]PIB retention in the frontal, parietal, temporal, and posterior cingulate areas under SPM analysis [38]. Furthermore, the averaged [^18^F]AV-45 SUVR distribution pattern in aMCI subjects positive for amyloidosis was more like Stage B by the Braak and Braak's amyloid deposition category, while the distribution pattern in aMCI subjects negative for amyloidosis was more like Stage A (Figure 2) [26]. Additionally, a more widely spread [^18^F]AV-45 uptake was noted over the frontal, temporal, and parietal areas in AD subjects than aMCI subjects in SPM analysis, which are concordant with those of the previous [^18^F]AV-45 and [^11^C]PIB studies [19], [39], [40]. Although the [^18^F]AV-45 distribution among the CN, aMCI and AD groups could mirror the pathological staging, its association with cognitive decline needs to be followed up in further cohort studies.

The deposition of amyloid plaques has been reported to occur at least 10 years prior to the clinical diagnosis of AD; about 21% of non-demented elderly have amyloid pathology [41]. However, the positivity of cerebral amyloidosis was relatively low in our CN group. Age is associated with amyloid load, and the relatively young age in our CN group may contribute to the low positivity [42].

Mild cognitive impairment is a transitional state between normal cognition and AD; it can be present years before the diagnosis of AD. The amyloid load in subjects with MCI is correlated with the rate of conversion from MCI to AD, suggesting that it is a prognostic marker in MCI subjects [27]. However, many clinical and genetic factors, such as age, APOE4 allele carrier, and cerebral atrophy severity, may also influence the amyloid load in MCI subjects [17], [42], [43]. Therefore, the distribution of amyloid load in MCI subjects could be diverse, and a great overlap with CN and AD groups can be expected [33], [44]. In our study, AD subjects tended to have a higher global cortical [^18^F]AV-45 SUVR (1.34±0.13) than aMCI patients (1.27±0.16) with an effect size of 0.45, but the sample size in this study is not large enough to differentiate the 2 groups based on the VOI analysis. Nonetheless, a more extensive [^18^F]AV-45 retention could be noted over the frontal, parietal, temporal and occipital areas under the voxelwise SPM analysis. On the other hand, amyloid deposition is an early change in the pathogenesis of AD. Rather than the cortical amyloid load, the downstream pathway such as synaptic dysfunction and brain structure change may play a more important role in determining the cognitive declines from MCI to AD [2], [27], and the application of multimodal neurochemical and imaging biomarkers should be considered to early identify predementia and even preclinical asymptomatic stages of AD [45].

Alzheimer's disease is characteristic of progressive amyloid plaques accumulation accompanied with cognitive decline. Roe *et al* found that mean cortical [^11^C]PIB binding potential was not only related to MMSE scores, but also the cognitive measures associated with executive functions among AD and CN subjects [46]. However, they didn't correlate the regional cortical [^11^C]PIB binding potential with the various cognitive measures. In the study by Barthel *et al*, both the frontal and total brain amyloid volumes on [^18^F]AV-1 PET were negatively correlated with MMSE scores among AD and CN subjects [47]. These findings are consistent with our study results that there was a negative correlation between MMSE scores and [^18^F]AV-45 SUVRs of various cortical regions, especially over the frontal, temporal and precuneus areas even after controlling for age and years of education. The presence of negative correlation between MMSE scores and the SUVRs of different amyloid radiotracers on PET may support the cascade hypothesis that amyloid plaque accumulation plays a pivotal role in the AD pathophysiological process [48].

There were several limitations in the current study. Correcting partial volume effect (PVE) is important for VOI-based analysis and in particular, for amyloid imaging. Therefore, the first limitation in this study is that no PVE correction was performed. The use of binary gray matter mask in this work is to avoid extracting activity from both CSF and white matter regions by confining VOI quantification only to gray matter region. To improve quantification accuracy in small VOIs, PVE correction should be included as a future work. Second, the sample size in our study is relatively small, and the results are not generalizable to the Chinese population. In addition, a cutoff value of 1.178 for neocortical [^18^F]AV-45 uptake was derived to differentiate Chinese AD from CN subjects, and its application to predict the conversion to AD from CN or aMCI subjects has to be verified in a longitudinal cohort study.

## Conclusions

The findings from our study show that [^18^F]AV-45 PET can be applied in vivo to differentiate the cerebral amyloid burden among CN, aMCI, and AD subjects using continuous or binary measurements, and [^18^F]AV-45 SUVR is associated with MMSE scores. The clinical diagnosis-based threshold for cerebral amyloidosis derived from AD patients is similar to the pathology-based threshold. Therefore, [^18^F]AV-45 PET is a potential biomarker for AD in multicenter monitoring and treatment trials.

## Supporting Information

Table S1 **Comparing [^18^F]AV-45 uptake between Alzheimer's disease (AD) patients and amnestic mild cognitive impairment (aMCI) patients.** The locations and values of the most significant increased [^18^F]AV-45 uptake in AD patients than aMCI patients, p<0.01 (unc.), extent voxels = 100.
(DOC)

Table S2 **Comparing [^18^F]AV-45 uptake between amnestic mild cognitive impairment (aMCI) patients and cognitively normal (CN) subjects.** The locations and values of the most significant increased [^18^F]AV-45 uptake in aMCI patients than CN subjects, p<0.01 (unc.), extent voxels = 100.
(DOC)

Table S3 **Comparing [^18^F]AV-45 uptake between Alzheimer's disease (AD) patients and cognitively normal (CN) subjects.** The locations and values of the most significant increased [^18^F]AV-45 uptake in AD patients than CN subjects, p<0.01 (unc.), extent voxels = 100.
(DOC)
