# Supplementary material for: Regional Amyloid Deposition in Amnestic Mild Cognitive Impairment and Alzheimer's Disease Evaluated by [18F]AV-45 Positron Emission Tomography in Chinese Population
Source: PLoS One. 2013 Mar 14;8(3):e58974. doi: 10.1371/journal.pone.0058974 (PMC3597555; doi:10.1371/journal.pone.0058974)
Supplement: Table S1 — Comparing [18F]AV-45 uptake between Alzheimer's disease (AD) patients and amnestic mild cognitive impairment (aMCI) patients. The locations and values of the most significant increased [18F]AV-45 uptake in AD patients than aMCI patients, p<0.01 (unc.), extent voxels = 100. (DOC) [file pone.0058974.s001.doc]

**Supporting information**

| **Table S1. Comparing [^18^F]AV-45 uptake between Alzheimer's disease (AD) patients and amnestic mild cognitive impairment (aMCI) patients.** The locations and values of the most significant increased [^18^F]AV-45 uptake in AD patients than aMCI patients, p<0.01 (unc.), extent voxels=100. | | | | | |
| --- | --- | --- | --- | --- | --- |
|  | | | | | |
| Brain region | Talairach coordinates | | | Brodmann  area | Z-score |
|  | x | y | z |  |  |
| L Middle Frontal Gyrus | -40 | -2 | 58 | 1 | 3.34 |
| L Middle Frontal Gyrus | -48 | 12 | 42 | 0 | 2.74 |
| L Middle Frontal Gyrus | -40 | -2 | 58 | 1 | 3.34 |
| L Middle Frontal Gyrus | -48 | 12 | 42 | 0 | 2.74 |
| R Middle Frontal Gyrus | 40 | -2 | 62 | 4 | 2.8 |
| R Middle Frontal Gyrus | 40 | -2 | 62 | 4 | 2.8 |
| R Middle Frontal Gyrus | 44 | 24 | 48 | 1 | 3.76 |
| L Superior Frontal Gyrus | -28 | 50 | 32 | 0 | 3.76 |
| L Superior Frontal Gyrus | -36 | 30 | 50 | 2 | 3.44 |
| L Superior Frontal Gyrus | -28 | 50 | 32 | 0 | 3.76 |
| L Superior Frontal Gyrus | -36 | 30 | 50 | 2 | 3.44 |
| L Superior Frontal Gyrus | -24 | 36 | 50 | 0 | 3.42 |
| L Superior Frontal Gyrus | -28 | 0 | 64 | 1 | 2.6 |
| R Inferior Frontal Gyrus | 60 | 14 | 18 | 0 | 3.27 |
| R Inferior Frontal Gyrus | 56 | 38 | 0 | 1 | 3.05 |
| R Inferior Frontal Gyrus | 60 | 14 | 18 | 0 | 3.27 |
| R Inferior Frontal Gyrus | 56 | 38 | 0 | 1 | 3.05 |
| R Inferior Frontal Gyrus | 54 | 34 | -8 | 1 | 2.93 |
| L Middle Occipital Gyrus | -50 | -76 | -4 | 0 | 2.73 |
| R Middle Occipital Gyrus | 44 | -80 | 12 | 1 | 2.8 |
| L Middle Temporal Gyrus | -48 | -76 | 16 | 1 | 3.46 |
| R Superior Temporal Gyrus | 64 | -2 | 10 | 1 | 2.69 |
| L Supramarginal Gyrus | -60 | -46 | 24 | 0 | 2.91 |
| L Cuneus | -20 | -92 | 24 | 0 | 2.67 |
| R Paracentral Lobule | 6 | -34 | 52 | 0 | 2.83 |
| R Postcentral Gyrus | 66 | -6 | 14 | 1 | 2.91 |
| R Postcentral Gyrus | 62 | -18 | 32 | 1 | 2.72 |
| R Postcentral Gyrus | 42 | -42 | 58 | 1 | 3.91 |
| R Postcentral Gyrus | 66 | -6 | 14 | 1 | 2.91 |
| R Precentral Gyrus | 52 | -6 | 46 | 0 | 3.81 |
| R Precentral Gyrus | 52 | -6 | 46 | 0 | 3.81 |
| R Precuneus | 26 | -84 | 40 | 3 | 3.7 |
| R Precuneus | 12 | -82 | 42 | 0 | 3.35 |
| R Precuneus | 26 | -84 | 40 | 3 | 3.7 |
| R Precuneus | 12 | -82 | 42 | 0 | 3.35 |
| L Cerebellum | -18 | -46 | -18 | 0 | 4.19 |
| L Cerebellum | -26 | -36 | -26 | 0 | 3.75 |
| R Cerebellum | 22 | -32 | -26 | 0 | 3.15 |
| R Cerebellum | 16 | -46 | -16 | 0 | 3.05 |
| R Cerebellum | 22 | -32 | -26 | 0 | 3.15 |
| R Cerebellum | 16 | -46 | -16 | 0 | 3.05 |
| R Cerebellum | 18 | -38 | -20 | 0 | 3.01 |
