# Supplementary material for: Regional Amyloid Deposition in Amnestic Mild Cognitive Impairment and Alzheimer's Disease Evaluated by [18F]AV-45 Positron Emission Tomography in Chinese Population
Source: PLoS One. 2013 Mar 14;8(3):e58974. doi: 10.1371/journal.pone.0058974 (PMC3597555; doi:10.1371/journal.pone.0058974)
Supplement: Table S2 — Comparing [18F]AV-45 uptake between amnestic mild cognitive impairment (aMCI) patients and cognitively normal (CN) subjects. The locations and values of the most significant increased [18F]AV-45 uptake in aMCI patients than CN subjects, p<0.01 (unc.), extent voxels = 100. (DOC) [file pone.0058974.s002.doc]

**Supporting information**

| **Table S2. Comparing [^18^F]AV-45 uptake between amnestic mild cognitive impairment (aMCI) patients and cognitively normal (CN) subjects.** The locations and values of the most significant increased [^18^F]AV-45 uptake in aMCI patients than CN subjects, p<0.01 (unc.), extent voxels=100. | | | | | |
| --- | --- | --- | --- | --- | --- |
|  | | | | | |
| Brain region | Talairach coordinates | | | Brodmann  area | Z-score |
|  | x | y | z |  |  |
| L Medial Frontal Gyrus | 0 | 38 | -10 | 2 | 4.28 |
| L Middle Frontal Gyrus | -44 | 44 | -14 | 0 | 4.22 |
| R Middle Frontal Gyrus | 40 | 58 | -18 | 4 | 4.62 |
| R Middle Frontal Gyrus | 40 | 34 | 44 | 2 | 4.55 |
| R Middle Frontal Gyrus | 38 | 42 | 36 | 0 | 4.29 |
| R Superior Frontal Gyrus | 28 | 40 | -22 | 4 | 4.94 |
| R Superior Frontal Gyrus | 38 | 52 | -22 | 5 | 4.39 |
| R Superior Frontal Gyrus | 24 | 40 | 46 | 0 | 4.25 |
| L Middle Temporal Gyrus | -48 | -74 | 16 | 2 | 4.24 |
| R Middle Temporal Gyrus | 56 | -64 | 16 | 0 | 4.53 |
| R Middle Temporal Gyrus | 56 | -26 | -4 | 2 | 4.37 |
| R Middle Temporal Gyrus | 60 | -36 | 2 | 0 | 4.32 |
| R Middle Temporal Gyrus | 46 | -76 | 24 | 2 | 4.26 |
| R Middle Temporal Gyrus | 64 | -4 | -12 | 0 | 4.25 |
| R Middle Temporal Gyrus | 56 | 6 | -38 | 5 | 4.23 |
| R Inferior Temporal Gyrus | 64 | -26 | -26 | 4 | 4.68 |
| R Inferior Temporal Gyrus | 66 | -22 | -24 | 4 | 4.46 |
| R Inferior Temporal Gyrus | 60 | -2 | -32 | 4 | 4.26 |
| R Superior Temporal Gyrus | 62 | -48 | 20 | 1 | 4.88 |
| R Superior Temporal Gyrus | 58 | -58 | 16 | 0 | 4.51 |
| R Superior Temporal Gyrus | 52 | 0 | 2 | 1 | 2.62 |
| R Superior Occipital Gyrus | 38 | -82 | 24 | 0 | 4.45 |
| L Superior Parietal Lobule | -32 | -46 | 60 | 0 | 3.08 |
| R Inferior Parietal Lobule | 52 | -64 | 40 | 1 | 4.94 |
| L Parahippocampal Gyrus | -22 | 2 | -16 | 1 | 2.65 |
| L Parahippocampal Gyrus | -24 | -8 | -18 | 0 | 2.43 |
| L Posterior Cingulate | -2 | -52 | 22 | 1 | 4.51 |
| L Lentiform Nucleus | -16 | 14 | -6 | 0 | 2.96 |
| R Cingulate Gyrus | 2 | 22 | 34 | 1 | 4.55 |
| R Insula | 42 | 8 | 2 | 0 | 2.75 |
| R Insula | 38 | 18 | 2 | 0 | 2.73 |
| R Orbital Gyrus | 22 | 34 | -26 | 2 | 4.79 |
| R Postcentral Gyrus | 28 | -30 | 58 | 0 | 2.96 |
| R Precuneus | 18 | -70 | 50 | 0 | 4.27 |
| R Precuneus | 38 | -80 | 36 | 0 | 4.23 |
| R Precuneus | 2 | -66 | 36 | 2 | 4.23 |
| L Cerebellum | -48 | -50 | -22 | 0 | 4.33 |
| R Cerebellum | 28 | -40 | -24 | 0 | 4.61 |
