# Supplementary material for: Regional Amyloid Deposition in Amnestic Mild Cognitive Impairment and Alzheimer's Disease Evaluated by [18F]AV-45 Positron Emission Tomography in Chinese Population
Source: PLoS One. 2013 Mar 14;8(3):e58974. doi: 10.1371/journal.pone.0058974 (PMC3597555; doi:10.1371/journal.pone.0058974)
Supplement: Table S3 — Comparing [18F]AV-45 uptake between Alzheimer's disease (AD) patients and cognitively normal (CN) subjects. The locations and values of the most significant increased [18F]AV-45 uptake in AD patients than CN subjects, p<0.01 (unc.), extent voxels = 100. (DOC) [file pone.0058974.s003.doc]

**Supporting information**

| **Table S3. Comparing [^18^F]AV-45 uptake between Alzheimer's disease (AD) patients and cognitively normal (CN) subjects.** The locations and values of the most significant increased [^18^F]AV-45 uptake in AD patients than CN subjects, p<0.01 (unc.), extent voxels=100. | | | | | |
| --- | --- | --- | --- | --- | --- |
|  | | | | | |
| Brain region | Talairach coordinates | | | Brodmann  area | Z-score |
|  | x | y | z |  |  |
| L Inferior Frontal Gyrus | -52 | 34 | 4 | 2 | 5.39 |
| L Inferior Parietal Lobule | -64 | -24 | 26 | 0 | 5.13 |
| L Inferior Temporal Gyrus | -66 | -24 | -20 | 2 | 5.3 |
| L Superior Frontal Gyrus | -42 | 18 | 52 | 0 | 5.89 |
| L Superior Frontal Gyrus | -30 | 52 | 28 | 0 | 5.23 |
| R Superior Frontal Gyrus | 40 | 18 | 54 | 2 | 6.09 |
| L Medial Frontal Gyrus | 0 | 42 | -12 | 2 | 5.29 |
| L Middle Frontal Gyrus | -40 | 26 | 46 | 0 | 5.89 |
| L Middle Frontal Gyrus | -34 | 44 | 36 | 0 | 5.87 |
| L Middle Frontal Gyrus | -38 | 30 | 46 | 0 | 5.83 |
| L Middle Frontal Gyrus | -44 | 18 | 48 | 1 | 5.56 |
| L Middle Frontal Gyrus | -44 | 48 | 14 | 1 | 5.23 |
| R Middle Frontal Gyrus | 40 | 30 | 46 | 0 | 6.02 |
| R Middle Frontal Gyrus | 38 | 40 | 40 | 1 | 5.96 |
| R Middle Frontal Gyrus | 32 | 40 | -22 | 4 | 5.16 |
| L Middle Occipital Gyrus | -50 | -74 | 0 | 0 | 5.6 |
| R Middle Occipital Gyrus | 48 | -76 | -8 | 0 | 5.63 |
| L Middle Temporal Gyrus | -48 | -76 | 14 | 1 | 5.86 |
| L Middle Temporal Gyrus | -56 | -66 | 8 | 0 | 5.47 |
| L Middle Temporal Gyrus | -58 | -62 | 8 | 1 | 5.37 |
| L Middle Temporal Gyrus | -60 | -58 | 10 | 1 | 5.15 |
| R Inferior Temporal Gyrus | 58 | -58 | -4 | 1 | 5.18 |
| L Paracentral Lobule | -8 | -46 | 58 | 0 | 5.33 |
| L Postcentral Gyrus | -62 | -20 | 32 | 0 | 5.1 |
| L Posterior Cingulate | 0 | -56 | 22 | 1 | 5.2 |
| L Precuneus | -2 | -52 | 34 | 0 | 5.25 |
| L Precuneus | 0 | -62 | 32 | 1 | 5.21 |
| R Precuneus | 16 | -62 | 20 | 2 | 5.16 |
| R Precentral Gyrus | 54 | -4 | 46 | 1 | 5.29 |
| L Cerebellum | -20 | -46 | -20 | 0 | 5.79 |
| R Cerebellum | 34 | -46 | -28 | 0 | 5.86 |
| R Cerebellum | 28 | -52 | -24 | 0 | 5.58 |
